# New Cryogels Based on Poly(vinyl alcohol) and a Copolymacrolactone System: I-Synthesis and Characterization

**DOI:** 10.3390/nano12142420

**Published:** 2022-07-14

**Authors:** Bianca-Elena-Beatrice Crețu, Loredana Elena Nita, Alexandru-Mihail Șerban, Alina Gabriela Rusu, Florica Doroftei, Aurica P. Chiriac

**Affiliations:** Department of Natural Polymers, Bioactive and Biocompatible Materials, Petru Poni Institute of Macromolecular Chemistry, 41 A Grigore Ghica Voda Alley, 700487 Iasi, Romania; cretu.bianca@icmpp.ro (B.-E.-B.C.); serban.alexandru@icmpp.ro (A.-M.Ș.); rusu.alina@icmpp.ro (A.G.R.); florica.doroftei@icmpp.ro (F.D.); achiriac@icmpp.ro (A.P.C.)

**Keywords:** poly(vinyl alcohol), ethylene brassylate, squaric acid, cryogels, wound dressing

## Abstract

Physical cryogels were obtained using the successive freeze–thaw technique of poly(vinyl alcohol) (PVA)/poly(ethylene brassylate-co-squaric acid) (PEBSA) solutions. The cryogel systems were prepared by using two different molecular weights of PVA and PEBSA with three different ratios between the ethylene brassylate (EB) and squaric acid (SA) comonomers. The presence of interactions, the thermal properties and the morphology were investigated using Fourier Transform Infrared Spectroscopy (FT-IR), thermogravimetry (TGA and DTG) and scanning electron microscopy (SEM), respectively. The influence of the composition on the degree of swelling in a physiological environment was demonstrated. The study highlighted improvements in terms of new network flexibility due to the intermolecular chains interactions brought by the introduction of PEBSA in the cryogel structure. We also concluded that the presence of PEBSA in the PVA/PEBSA cryogel network improved the loading capacity of the new system with specific hydrophobic agents, for example essential oils, which (due to their antimicrobial character) can lead to the use of new systems obtained for various applications.

## 1. Introduction

Hydrogels are three-dimensional networks made up of hydrophilic polymer chains, capable of swelling, absorbing and retaining impressive amounts of water [1]. The water retention characteristic is due to the presence of the specific groups of the polymer chains (–OH, –COOH, –NH_2_, –CONH_2_ and –SO_3_H) able to establish hydrogen bonds with water without dissolving [2]. In terms of molecular interactions at the molecular level, elasticity and high water content, hydrogels are similar to natural biological tissues.

Among the synthetic polymers, poly(vinyl alcohol) (PVA) is one of the most used polymers for obtaining hydrogels with various applicability from biomedical field to intelligent materials or water treatment [3,4,5,6,7,8,9,10].

PVA is a water-soluble polymer that is biocompatible, biodegradable and non-toxic and offers a wide range of applications. Due to its hydroxyl groups, it allows changes in surface and volume properties [3]. Despite the above properties, the use of pure PVA-based hydrogels as biomaterial is limited by the absence of bioactivity and insufficient mechanical strength [4]. Obtaining physically crosslinked PVA hydrogels through repeated freeze–thaw method occurs due to the molecular structure and strong tendency of PVA solutions to crystallize [5]. The properties of the cryogel depend on the average molecular weight and the degree of hydrolysis of PVA, the concentration of polymer in the solution, the number of freeze–thaw cycles, cycle duration and the temperature used.

The chemical structure of PVA allows modification reactions that can be regarded as a main tool for adjusting the final properties of the cryogel [6]. The shortcomings mentioned above can be balanced by the presence of other polymers. By combining different kinds of polymers with various characteristics, new systems with improved and superior properties can be generated. Blends of PVA with synthetic or natural polymers are the simplest and most common method of modifying the physico-chemical properties of the material, such as the elasticity, morphology, porosity, swelling capacity, thermal stability and mechanical properties [7,8,9,10].

Our group reported recently the synthesis of poly(ethylene brassylate-co-squaric acid) (PEBSA) performed by ethylene brassylate macrolactone ring-opening and copolycondensing with squaric acid [11]. In previous studies, our group paid particular attention to this copolymeracrolactone system with different ratios between comonomers [11,12,13]. PEBSA, which presents special properties, such as the possibility to create networks, biodegradability and good thermal stability, is also capable of incorporating essential oils—namely thymol (Thy) and carvacrol (CC). The polymeric system allowed the entrapment of bioactive compounds through a procedure of inclusion complexation due to its amphiphilic character. Moreover, the hydrophobic affinity of the compounds involved in the system led to a good dispersion of the bioactive molecular compounds in the PEBSA polymer network.

The present study investigates the possibility of a new cryogel preparation as a network system with increased capacity for subsequent incorporation of hydrophobic compounds. The paper presents the synthesis of new PVA/PEBSA cryogel systems, their characterization in terms of structural and thermal properties and the influence of the composition on the degree of swelling as well as the morphology of the network. It is intended that the new system can be used for the incorporation of hydrophobic bioactive agents, such as essential oils, which (due to their antimicrobial character) can lead to the use of the new synthesized network for different applications, such as wound dressing and food packaging.

## 2. Materials and Methods

### 2.1. Materials

Ethylene brassylate (EB, 1,4-dioxacycloheptadecane-5,17-dione, C_15_H_26_O_4_, Mw = 270.36 g/mol, purity of 95.0%), squaric acid (SA, 3,4-dihydroxy-3-cyclobutene-1,2-dione, H_2_C_4_O_4_, Mw = 114.06 g/mol, purity > 99.0%) and 1,4-Dioxane (purity ≥ 99.0%) were purchased from Sigma-Aldrich (Darmstadt, Germany). Poly(vinyl alcohol) (PVA, with different molecular weights, 72,000 and 145,000 g/mol) was acquired from Merck (Hohenbrunn, Germany), and anhydrous 1-hexanol was from Across-Organics (Geel, Belgium). All chemicals were used as received without further purification.

### 2.2. Method

#### Preparation of Cryogels

Poly(ethylene brassylate-co-squaric acid) (PEBSA) was synthetized from ethylene brassylate and squaric acid by the ring-opening copolymerization procedure described before [11]. The formation of the cryogels from two PVA variants of different molecular weights, namely 72,000 and 145,000 g/mol, and PEBSA with different ratios between EB/SA comonomers, namely 25/75, 50/50 and 75/25, was realized by individually preparing of the five stock solutions and by mixing them in different volumetric ratios (Table 1).

Briefly, 4% *w/v* PVA solutions were prepared by the dissolution of appropriate amounts of PVA granules in distilled water and magnetic stirring at 90 °C over a period of 2 h. PEBSA (4% *w/v*) solution was prepared by mixing an appropriate amount of copolymer in 1,4-dioxane and magnetically stirring at room temperature. Both solutions were mixed together at different ratios (Table 1), poured into molds and then subjected to three consecutive freeze–thaw cycles of 18 h freezing at −20 °C followed by thawing for 8 h at 25 °C (ambient temperature). Subsequently, the prepared cryogels were lyophilized for the characterization. After these freeze–thaw cycles, the gels formed were visually examined, and we found that the most uniform, consistent gels that had formed in the whole mass of liquid were recorded at a ratio of 2/1. These are the variants that are characterized in the following part.

### 2.3. Characterization

#### 2.3.1. FT-IR

Fourier Transform Infrared Spectroscopy (FT-IR) spectra of the cryogels were obtained using a Vertex 70 Spectrometer from Bruker (Ettlingen, Germany). Samples were prepared using the potassium bromide disk method: 1 mg of dried sample was mixed with potassium bromide powder, grinded and then compressed into disk shape. The spectra were obtained in transmittance mode in the range of wavenumber from 4000 to 400 cm^−1^ with a resolution of 4 cm^−1^ and an average of 64 scans.

#### 2.3.2. Thermal Analysis

To characterize the thermal behavior of PVA as well as PVA_PEBSA cryogel samples, thermogravimetric analysis was performed on a Jupiter STA 449 F1 thermobalance produce by Netzsch (Selb, Germany). Freeze dried samples with a weight between 10 and 12 mg was placed in Al_2_O_3_ crucibles and heated from room temperature up to 675 °C in dynamic mode with a heating rate of 10 °C/min and 40 mL/min nitrogen flow. The data obtained were subsequently processed using Proteus Analysis software.

#### 2.3.3. SEM Microscopy

The cross-section SEM micrographs of the samples were obtained with a Quanta 200 electron microscope (from FEI Company, Hillsboro, OR, USA), working at 20 kV in low vacuum mode, without any coating. The samples were fixed on aluminum stubs with double-adhesive carbon tape. The average pore size was calculated using ImageJ software.

#### 2.3.4. Swelling Studies

The swelling behavior of freeze-dried cryogels was studied in phosphate buffer solution (0.01 M, pH = 5.4) at 25 °C (ambient temperature) using the gravimetric method. At different intervals of time, the weight of the samples was noted after collecting from the buffer solution and blotting the surface liquid excess with filter paper. The measurements were continued until equilibrium swelling was reached. The swelling degree was calculated based on the following formula:

$$\mathrm{Swelling}\;\mathrm{degree}=\frac{W_{t}-{\;W}_{0}}{W_{0}}\times 100$$

where W_t_ is the weight of the swollen cryogels at a particular time t, and W_0_ is the initial weight of the dry samples (g).

## 3. Results and Discussion

**Figure 1:**
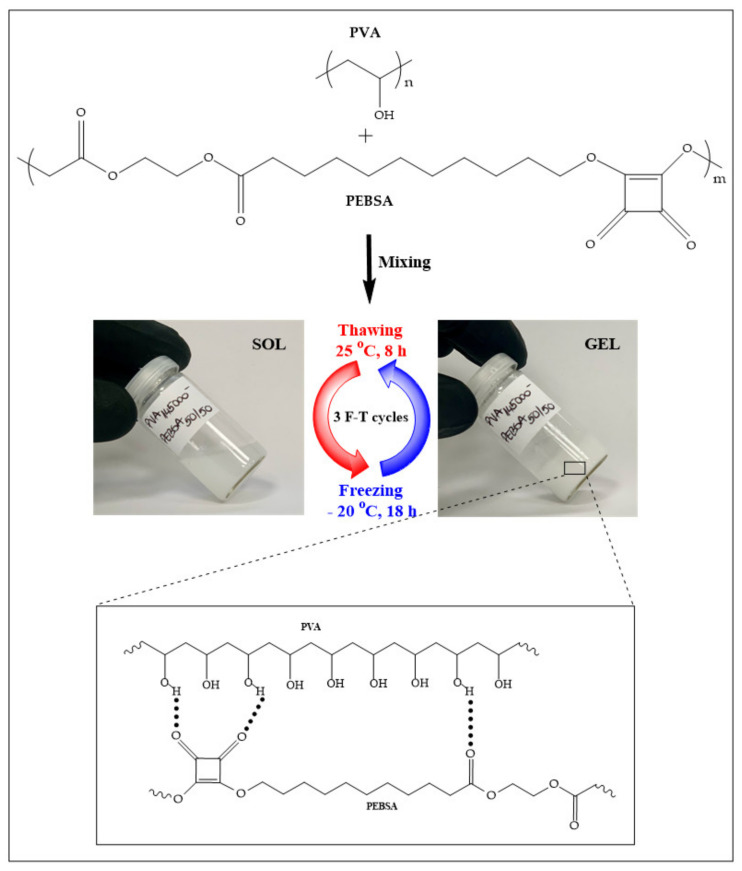
Schematization of the new cryogel structure based on PVA and PEBSA copolymacrolactone.

The study highlights the possibility of preparing a new cryogel structure based on a PVA and PEBSA copolymacrolactone blend, as illustrated in Figure 1, and to what extent the presence of PEBSA (designated for easier coupling in the network of hydrophobic compounds) modifies the properties of the polymer matrix.

### 3.1. FT-IR

**Figure 2:**
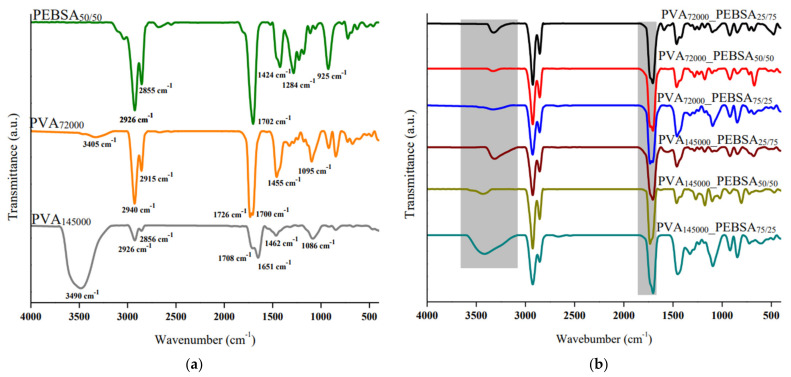
(**a**) PEBSA_50/50_, PVA_72000_ and PVA_145000_ cryogel; and (**b**) PVA_72000__PEBSA_25/75_, PVA_72000__PEBSA_50/50_, PVA_72000__PEBSA_75/25_, PVA_145000__PEBSA_25/75_, PVA_145000__PEBSA_50/50_ and PVA_145000__PEBSA_75/25_ cryogel spectra.

The FT-IR spectra of the investigated samples presented in Figure 2 confirm the presence of the specific chemical groups as well as the chemical structure of the polymer pair involved in the new gel network formation.

Cryogels spectra contain both PEBSA and PVA specific bands and also changes that confirm the blend preparation and the presence of intermolecular bonds between PVA and PEBSA. The most important changes occurring in the cryogels spectra are registered in the region of 3700–3000 cm^−1^, at the level of O–H stretching vibration. The presence of hydroxyl stretching vibration shifted to lower values, from 3405 and 3490 cm^−1^ for PVA_7200_ and PVA_145000_, respectively, to 3327 cm^−1^ for PVA_7200_ blends and 3315–3343 cm^−1^ for PVA_145000_ blends, with variable intensities in all cryogel spectra, which is attributed to the interaction between the two polymers and formation of intermolecular bonds. 

Moreover, the reducing in intensity of C=O stretching vibration (~1700 cm^−1^) and C–O stretching vibration bands (~1280 cm^−1^) corresponding to PEBSA, settle the above statement. Other bands identified in spectra are: 2925, 2856 and 1460 cm^−1^ assigned to C–H asymmetric, symmetric and bending vibration; peaks found between 1096 and 1105 cm^−1^ correspond to asymmetric C–O–C bridge stretching vibration; and C=C bending vibration from squaric acid units identified at 920 and 848 cm^−1^ [13].

Considering all previous observations, we can conclude that physically crosslinked cryogels were successfully synthesized. Between the samples, differences appear in term of intensities and frequencies, and no new bands were observed. The lowest values of the O–H tensile vibration band were recorded for the PEBSA_25/75_ samples—values that are due to the greater number of units squaric acid units available for intermolecular hydrogen bond formation.

### 3.2. Thermal Characterization

**Figure 3:**
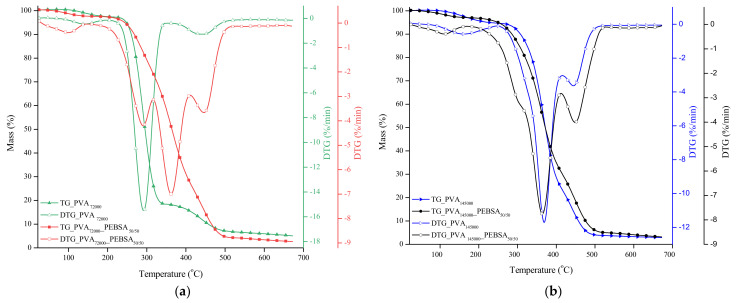
(**a**) The TG and DTG curves of the PVA_72000__PEBSA_50/50_ cryogel and (**b**) the TG and DTG curves of the PVA_145000__PEBSA_50/50_ cryogel.

The behavior of the investigated samples with heat treatment is illustrated in Figure 3. Thus, the thermogravimetric and first derivative thermogravimetric curves of PVA_72000_ and PVA_72000__PEBSA_50/50_ cryogel sample are presented in Figure 3a, whereas Figure 3b presents the thermogravimetric and first derivative thermogravimetric curves of PVA_145000_ and PVA_145000__PEBSA_50/50_ cryogel samples. Table 2 presents the values of the principal thermal parameters registered for the investigated samples during the thermal treatment.

Both PVA samples presented three degradation stages of thermal decomposition process. The first stage, which begins at about 90 °C and reaches the maximum degradation rate around 140 and 162 °C, presents the loss of weakly physically absorbed water and decomposition of water and acetaldehyde vapors [14].

The second stage of the process is mainly ascribed to the dehydration reactions, thermal degradation reactions resulting in random chain scission and the formation of high molecular polyene structures. Unlike the first stage, this stage took place at different temperature ranges, depending on the sample: between 248 and 396 °C for PVA_72000_, and between 290 and 422 °C for PVA_145000_. The third stage of degradation is found at higher temperatures, above 400 °C, and this is assigned to further degradation of the intermediary polyene structure into small molecular weight compounds via chain-scission reactions [15]. If we refer to thermal stability, the T_10_ and T_20_ parameters must be considered. 

As presented in Table 2, PVA with higher molecular mass (145,000) has better thermal stability with a higher T_onset_ of all stages. This behavior is attributed to several factors, such as the higher crystallinity, higher capacity to form intramolecular hydrogen bonds and a more organized supramolecular structure. All data collected are in good correlation with the literature in the field [16,17].

According to our investigations, the PEBSA variants have two to three stages of thermal degradation depending on the molar ratio between the comonomers, and start from 260 °C to a maximum degradation temperature of 460 °C [12]. Considering that, in previous studies [12], the thermal analysis was performed on all three variants of copolymers, with the studies showing the appearance of differences only in the case of PEBSA_50/50_, in this paper, this variant was studied in thermal analysis of the obtained cryogels.

PVA/PEBSA mixtures allow the formation of cryogels that have four stages of thermal degradation that are much more clearly individualized in the case of thePVA_72000__PEBSA_50/50_ sample. The first degradation stage, which begins at low temperatures, 32 and 38 °C, respectively, is caused by the evaporation of the water physically absorbed on the samples structure. 

The next degradation stage, assigned to PVA specific dehydration reactions, manifests differently for each sample: the PVA_72000_ blend presents a well-defined stage, which begins at 235 °C and reaches the maximum degradation rate at 293 °C, while this stage appears as a shoulder-stage with his onset temperature at 267 °C for the PVA_145000_ blend, right before the beginning of the third stage. The third stage is the main degradation stage from the process. This stage identifies with PEBSA copolymer backbone degradation process during which CO, CO_2_ and H_2_O are released. The last degradation stage is assigned to further degradation of the intermediary polyene structure and degradation of ethylene brassylate units.

For the PVA_72000_ blend with PEBSA, the presence of copolymacrolactone materializes by enhancing the thermal stability of the system, which is confirmed by the T_10_ and T_20_ parameters values. Another significant change appears at the level of the main degradation stage where the T_onset_ and T_peak_ values increased to 80 and 72 °C, respectively. On the other hand, for PVA_145000__PEBSA_50/50_ cryogels, the presence of PEBSA is concretized in a slightly decrease of the thermal stability as revealed by the T_10_ and T_20_ values.

This can be explained as it follows: the less ordered, relaxed PVA_72000_ supramolecular structure can interact more with PEBSA_50/50_ molecules, creating new hydrogen bond and thus a network with higher thermal stability. In contrast, the more crystalline, compact and ordered PVA_145000_ structure with numerous intramolecular hydrogen bonds is slightly affected by the PEBSA_50/50_ addition from thermal point of view. The above statement is also supported by the different characteristics of the second degradation stage of both cryogels.

Summarizing, the blend between PVA and PEBSA_50/50_ produces systems with enhanced thermal stability in case of the PVA variant with lower molecular mass.

### 3.3. SEM Studies

**Figure 4:**
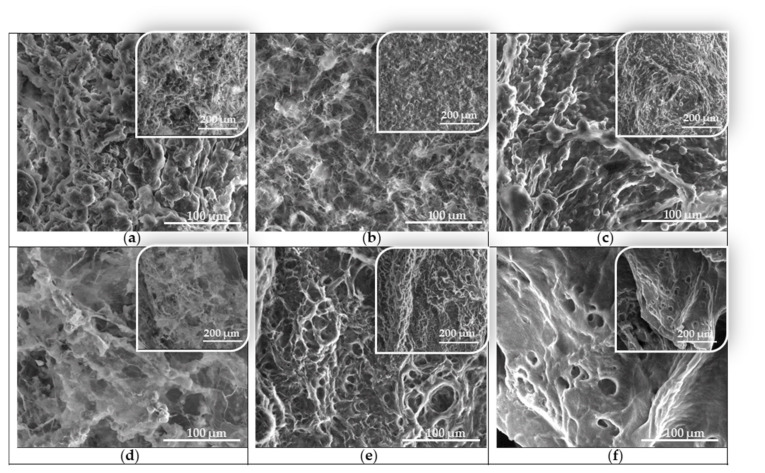
Scanning electron microscopy images of hydrogels at two different magnifications: (**a**) PVA_72000__PEBSA_25/75_, (**b**) PVA_145000__PEBSA_25/75_, (**c**) PVA_72000__PEBSA_50/50_, (**d**) PVA_145000__PEBSA_50/50_, (**e**) PVA_72000__PEBSA_75/25_ and (**f**) PVA_145000__PEBSA_75/25_ cryogels.

The SEM images of the synthesized variants of cryogels based on PVA and PEBSA illustrate their tridimensional network consisting of porous structures with interconnected pores (Figure 4). We also highlight the correlation between the conditions of cryogel preparation mentioned in Table 1—namely the molecular weight of the PVA and the ratio between EB and SA in the PEBSA system and the revealed images by SEM.

Thus, as shown in Figure 4, all hydrogel samples had a high porosity with almost spherical pores. The morphologies of the hydrogels were different in the sense that the pore structures differ from one sample to another by the thickness of the pore walls as well as by their size. Even though all of them have pores of a few hundred nanometers up to 2–10 μm in diameter, their thickness also varies depending on the nature of the hydrogel components. 

Therefore, in the case of the PVA_72000__PEBSA sample (Figure 4a,c,e), with the increase of the EB/SA ratio in the sample, the homogeneity and the pore size increases. This is to be expected, given that, as the content of the SA increases, so does the number of intermolecular bonds between the blend partners. In the case of Figure 1a, the image of PVA_72000__PEBSA_25/75_ cryogel structure, nano- and micrometric pores can be observed (from 400 nm to 2–3 μm). The pore walls are well contoured and thicker compared to samples c and e. 

**Figure 5:**
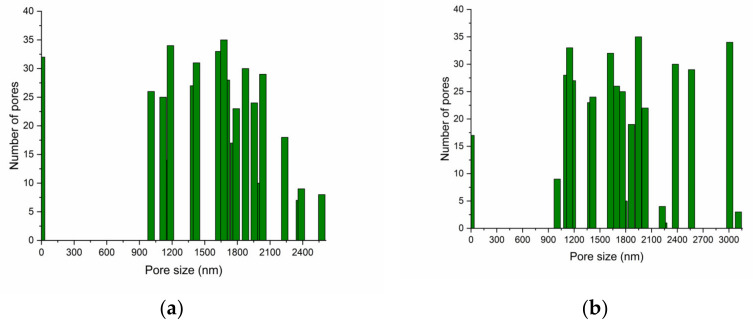
Average pore size calculated using ImageJ software for (**a**) PVA_72000__PEBSA_50/50_ and (**b**) PVA_145000__PEBSA_50/50_.

The morphology of the PVA_145000__PEBSA hydrogels can be observed in Figure 4b,d,f. In this case, with the increase of the EB/SA ratio, the degree of homogeneity of the samples decreases. Thus, in the case of sample b, the pores have dimensions of approximately 1.5–2 μm, and in the case of sample f, there are both pores with dimensions of tens of nanometers but also numerous pores of several hundred nanometers as confirmed by the average pore size calculated using ImageJ software (Figure 5).

These differences are associated with the increase in the number of carbonyl functional groups and resulting in growth of hydrogen bonds number [12]. Thus, the pore size and shape of PVA/PEBSA cryogels can be modified by adjusting the ratio between EB and SA comonomers from PEBSA composition, thus, providing the flexibility in the preparation of cryogels with the desired properties for the targeted application.

**Figure 6:**
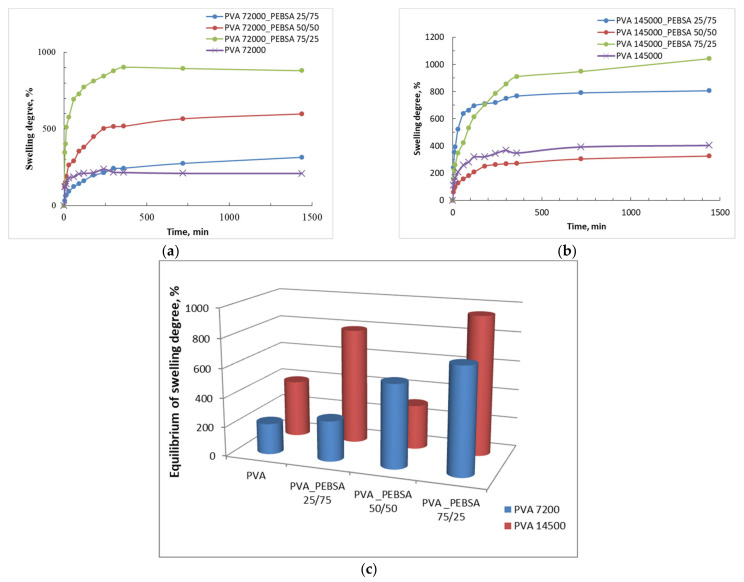
Degree of swelling vs. immersion time (min) for different composition PVA/PEBSA cryogels: (**a**) Mw_PVA_ = 72,000 Da, (**b**) Mw_PVA_ = 145,000 Da and (**c**) comparison between the samples regarding the maximum swelling degree.

The morphological characterization of the studied cryogel samples is in good agreement with swelling behavior as a function of time (Figure 6) and the ability to incorporate and release the bioactive compounds.

### 3.4. Degree of Swelling

The process of swelling is based on the penetration of the fluid molecules into the interior of the immersed polymeric network determining the pores to expand. The high swelling ability of the cryogel is preferable for wound dressing as such dressings can effectively protect the wound bed from exudate accumulation, thus, reducing the risk of infection as well as providing a moist environment. Due to the macroporous structure and hydrophilic nature of the gels, water can easily enter the pores to effectively swell the cryogels [16]. The equilibrium swelling ratios of the prepared cryogels have been shown to be influenced by their composition, thereby, resulting in higher swelling ratios for the flexible pores that facilitate the expansion of the network as illustrated in Figure 6.

All the investigated samples presented a rapid swelling rate in the first minutes, followed by a slower process until reaching a plateau. Cryogels based only on PVA, which are more compact, presented a lower degree of swelling. The presence of PEBSA determines the formation of the network with larger meshes, leading to an increase of the degree of swelling. The exception is the sample PVA_145000__PEBSA_50/50_, which is attributed to a more orderly and compact structure with more physical crosslinks between PVA hydroxyl groups and SA carbonyl groups that limits the access of the solvent in the meshes of the network. 

Therefore, the minimum swelling capacity was recorded for the sample containing PVA_72000_ and the large amount of SA comonomer (PVA_72000__PEBSA_25/75_). Increasing the amount of SA comonomer to 75% in the copolymer chemical structure induced some compaction due to the increase in the number of carbonyl functional groups and resulting of strong hydrogen bonds that induced a reduced swelling capacity. The smaller amount of SA comonomer (25%) determined the formation of a dense polymeric network with a smaller pore size, which led to the highest degree of swelling corresponding to the PVA_145000__PEBSA_75/25_ sample at a maximum value of 1048.57%. The swelling of the prepared cryogels was also found to increase with the increase the PVA molecular weight. This is in correlation with the SEM microscopy that attests, in this case, the formation of a more relaxed network with higher meshes.

## 4. Conclusions

The study confirmed the possibility of preparing a cryogel based on PVA and PEBSA obtained through the repeated freeze–thaw process and formation of physical intermolecular bonds between the polymeric pair. The properties of cryogels depend on the composition of the cryogels—namely the molecular weight of the PVA and the ratio between the EB and SA comonomers of the copolymacrolactone system. The chemical structure of the cryogels was confirmed by the spectroscopic method. 

The characteristic peaks from FT-IR spectra underlined the presence of intermolecular hydrogen bonds between the hydroxyl groups of PVA units and the carbonyl groups of SA units from PEBSA structure. The thermogravimetric analysis demonstrated that the incorporation of PEBSA_50/50_ in the PVA structure of the sample determined the increase of the thermal stability of the system. The SEM images of freeze-dried cryogels demonstrated that the tridimensional networks consist of porous structures with interconnected nano-sized pores. 

SEM analysis also confirmed the important role of the system composition in obtaining cryogel structures with the desired properties. The study also underlined the dependence of the swelling properties of the prepared networks on the presence of copolymacrolactone in the structure of the cryogel. The introduction of PEBSA determined the formation of a network with appropriate meshes, leading to increased swelling. The swelling of cryogels was found to increase with the increasing of the molecular weight of PVA, which is also in correlation with the SEM microscopy, thereby, attesting the formation of more relaxed networks.

## Figures and Tables

**Table 1:** Sample names and methods of preparation.

Sample *	PVA/PEBSA Ratio	Notation	Observations **	Photographs of PVA/PEBSA Cryogels
PVA_72000__PEBSA_25/75_	3/1	PVA_72000__PEBSA_25/75__3/1	Compact and opalescent gel	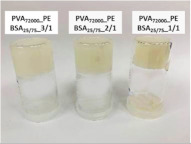
	2/1	PVA_72000__PEBSA_25/75__2/1	Compact gel	
	1/1	PVA_72000__PEBSA_25/75__1/1	Compact and strong gel	
PVA_145000__PEBSA_25/75_	3/1	PVA_145000__PEBSA_25/75__3/1	Compact and opalescent gel	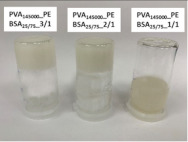
	2/1	PVA_145000__PEBSA_25/75__2/1	Compact gel	
	1/1	PVA_145000__PEBSA_25/75__1/1	Compact and strong gel	
PVA_72000__PEBSA_50/50_	3/1	PVA_72000__PEBSA_50/50__3/1	Compact and opalescent gel	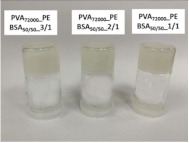
	2/1	PVA_72000__PEBSA_50/50__2/1	Compact gel	
	1/1	PVA_72000__PEBSA_50/50__1/1	Compact and strong gel	
PVA_145000__PEBSA_50/50_	3/1	PVA_145000__PEBSA_50/50__3/1	Compact and opalescent gel	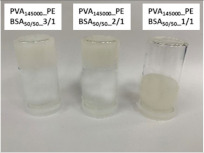
	2/1	PVA_145000__PEBSA_50/50__2/1	Compact gel	
	1/1	PVA_145000__PEBSA_50/50__1/1	Compact and strong gel	
PVA_72000__PEBSA_75/25_	3/1	PVA_72000__PEBSA_75/25__3/1	Compact and opalescent gel	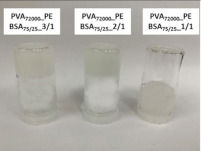
	2/1	PVA_72000__PEBSA_75/25__2/1	Compact gel	
	1/1	PVA_72000__PEBSA_75/25__1/1	Compact and strong gel	
PVA_145000__PEBSA _75/25_	3/1	PVA_145000__PEBSA_75/25__3/1	Compact and opalescent gel	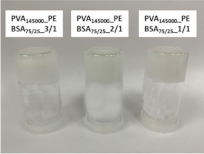
	2/1	PVA_145000__PEBSA_75/25__2/1	Compact gel	
	1/1	PVA_145000__PEBSA_75/25__1/1	Compact and strong gel	

*—4% *w/v* concentration for both polymer solutions. **—all variants of the polymer solutions mixture led to the formation of gels.

**Table 2:** The main thermal parameters determined for the cryogel samples.

Sample	Degradation Stage	T_onset_ (°C)	T_peak_ (°C)	W (%)	Residue (%)	T_10_ (°C)	T_20_ (°C)
PVA_72000_	I	90	140	2.55	5.05	260	273
	II	248	293	79.5			
	III	397	442	12.9			
PVA_145000_	I	89	162	5.27	2.88	309	336
	II	290	370	70.01			
	III	423	445	21.84			
PVA_72000__PEBSA_50/50_	I	38	105	2.76	2.63	268	296
	II	235	293	25.8			
	III	328	365	45.63			
	IV	422	449	23.53			
PVA_145000__PEBSA_50/50_	I	32	117	2.94	3.17	286	319
	II	267	305	16.63			
	III	330	365	49.85			
	IV	421	449	27.41			

## Data Availability

Not applicable.
